# Anesthesiologists ultrasound-guided regional anesthesia core curriculum: a Delphi consensus from Italian regional anesthesia experts

**DOI:** 10.1186/s44158-024-00190-2

**Published:** 2024-08-10

**Authors:** Alessandro De Cassai, Astrid Behr, Dario Bugada, Danilo Canzio, Gianluca Capelleri, Fabio Costa, Giorgio Danelli, Grazia De Angelis, Romualdo Del Buono, Fabrizio Fattorini, Pierfrancesco Fusco, Fabio Gori, Alberto Manassero, Ilaria Pacini, Giuseppe Pascarella, Mauro Proietti Pannunzi, Gianluca Russo, Raffaele Russo, Domenico Pietro Santonastaso, Marco Scardino, Giuseppe Sepolvere, Paolo Scimia, Alessandro Strumia, Mario Tedesco, Andrea Tognù, Vito Torrano

**Affiliations:** 1 Anesthesia and Intensive Care Unit “Sant’Antonio”, University Hospital of Padua, Padua, Italy; 2 Department of Anesthesiology and Intensive Care, Camposampiero Hospital, ULSS 6 Euganea Padova, Camposampiero, Italy; 3 Department of Emergency and Intensive Care, ASST Papa Giovanni XXIII, Bergamo, Italy; 4 Department of Anesthesia and Intensive Care Unit and Pain Therapy, Mater Dei Hospital, Bari, Italy; 5 Anesthesia, Intensive Care and Pain Therapy, Policlinico Di Monza, Monza, Italy; 6 Unit of Anesthesia, Intensive Care and Pain Management, Campus Bio-Medico University Hospital Foundation, Rome, Italy; 7 GVM Care and Research Maria Cecilia Hospital, Cotignola, Italy; 8 IRCCS Casa Sollievo Della Sofferenza, Foggia, Italy; 9 Unit of Anesthesia, Intensive Care and Pain Management, ASST Gaetano Pini, Milan, Italy; 10 Anaesthesiology, Critical Care Medicine and Pain Therapy, “Sapienza” University of Rome, Rome, Italy; 11 Department of Anesthesia, Intensive Care and Pain Medicine, SS. Filippo E Nicola Hospital, Avezzano, L’Aquila Italy; 12 University Hospital Santa Maria Della Misericordia, Udine, Italy; 13 Unit of Anesthesia, Casa Di Cura Città Di Bra, Cuneo, Italy; 14 Unit of Anaesthesia and Pain Therapy, Department of Obstetrics, Gynecology and Pediatrics, Sant’Orsola-Malpighi University Hospital, Bologna, Italy; 15 Casa Di Cura Villa Dei Pini, Civitanova Marche, Italy; 16 Anesthesia and Intensive Care, ASST Lodi, Lodi, Italy; 17 IRCCS, Casa Sollievo Della Sofferenza, San Giovanni Rotondo, Italy; 18 Anesthesia Unit, Ospedale Bufalini, Cesena, Italy; 19 Ortho Center, Humanitas Research Hospital, Milan, Italy; 20 Department of Anesthesia and Cardiac Surgery Intensive Care Unit, San Michele Hospital, Maddaloni, Caserta, Italy; 21 Department of Anesthesia and Intensive Care Unit, G. Mazzini Hospital, Teramo, Italy; 22 Department of Anesthesia, Critical Care and Pain Medicine, ASST Grande Ospedale Metropolitano Niguarda, Milan, Italy

**Keywords:** Regional anesthesia, Core curriculum, Residents, Education, Standardize, Training

## Abstract

**Introduction:**

The need for a standardized core curriculum in regional anesthesia has become essential, particularly with the integration of ultrasound revolutionizing and exponentially increasing clinical practice and possibilities. In fact, numerous novel techniques, often overlapping, can confuse practitioners. This study aims to establish a core curriculum for upper limb, lower limb, paraspinal and fascial plane blocks for residency training, addressing potential educational gaps caused by the multitude of techniques, through a Delphi consensus process involving recognized Italian regional anesthesia experts.

**Methods:**

A steering committee was formed in order to select a panel of experts in regional anesthesia. A three-round Delphi consensus was planned: two rounds of electronic voting and a final round of mixed electronic voting and round table discussion. The consensus was defined as ≥ 75% agreement for inclusion and lower than ≤ 25% agreement for exclusion from the core curriculum list. Techniques reaching the 50% threshold were included with low consensus.

**Results:**

Twenty-nine techniques were selected to be included in the ultrasound-guided regional anesthesia core curriculum. Twenty-two were included with strong consensus:

- *Upper limb*: interscalene brachial plexus block, supraclavicular brachial plexus block, infraclavicular brachial plexus block, axillary brachial plexus block, intermediate cervical plexus block
- *Lower limb*: femoral nerve block, pericapsular nerve group block, adductor canal block, sciatic nerve block (transgluteal approach, infragluteal approach, and at the popliteal fossa), ankle block
- *Paraspinal/fascial plane blocks*: erector spinae plane block, deep serratus anterior plane block, superficial pectointercostal plane block, interpectoral plane block, pectoserratus plane block, rectus sheath block, ilioinguinal iliohypogastric nerves block, transversus abdominis plane block (with subcostal and midaxillary approaches)

The remaining seven techniques were included with low consensus: superficial cervical plexus block, lumbar plexus block, fascia iliaca block (suprainguinal approach), anterior quadratus lumborum block, lateral quadratus lumborum block, paravertebral block, and serratus anterior plane block.

**Conclusions:**

This curriculum aims to standardize training and ensure that residents acquire the essential skills required for effective and safe practice regardless of the residents’ subsequent specialization. By incorporating these techniques, educational programs can provide a structured and consistent approach to regional anesthesia, enhancing the quality of patient care and improving outcomes.

**Supplementary Information:**

The online version contains supplementary material available at 10.1186/s44158-024-00190-2.

## Introduction

In the evolving landscape of medical education, particularly in the field of regional anesthesia, the need for a standardized and comprehensive core curriculum has become necessary.

In fact, the integration of ultrasound into clinical practice has indeed revolutionized how anesthesiologists approach patient care, enabling precise placement of needles inside fascia layers or in close proximity to small nerves [1].

In particular, in the last few years, we saw an increase of interest in describing novel approaches to regional anesthesia [2, 3]; however, often these techniques are similar or even overlapping and the resulting nomenclature could be confusing for practitioners [4].

In recent years, scientific societies specifically interested in regional anesthesia, namely European Society of Regional Anesthesia (ESRA) and American Society of Regional Anesthesia (ASRA) have collaboratively worked together in order to standardize the nomenclature for regional anesthesia techniques focusing on the upper limb, lower limb, and paraspinal and fascial plane blocks [5, 6].

While the novel-approved nomenclature is not yet widespread in the scientific literature [7], it undeniably serves as the cornerstone for developing scientific and educational resources.

However, with the multitude of regional anesthesia techniques potentially overshadowing educational opportunities during residency, it becomes imperative to establish a core curriculum for residents, regardless of their subsequent specialization.

In this framework, the aim of the present study was to determine the components of a core curriculum for residency training through a Delphi consensus process among Italian regional anesthesia recognized experts.

## Methods

A steering committee was formed (ADC, VT, PF, FC) to define the aims, the timeline, and the methodology, to set the agenda for the Delphi rounds, and to define the panel.

According to previous literature, a modified Delphi methodology was chosen as it is a validated process to achieve consensus regarding a specific topic among experts [8]. In fact, this method is renowned for its iterative and consensus-building approach, and serves as an ideal mechanism to navigate the complexities inherent in developing a core curriculum facilitating the evaluation of diverse opinions, resolving disagreements, and ultimately fostering agreement [9].

### *Panel selection*

Each member of the steering committee (ADC, FC, VT, PF) independently compiled a list of Italian experts in the field of regional anesthesia, considering scientific profiles and clinical experience. The final panel of experts was then determined by comparing the various lists, ensuring that an individual was nominated by at least two committee members to qualify as an expert for this study to minimize the risk of establishing personal viewpoints as consensus.

### List of techniques

A comprehensive literature search was performed to retrieve the most recent nomenclature for regional anesthesia techniques, and the search strategy is available as Supplementary Digital Content 1. Then, a list of regional anesthesia techniques for upper and lower limbs and paraspinal and fascial plane blocks was created using the recent ASRA/ESRA consensus documents regarding upper and lower limb and paraspinal and fascial plane blocks [5, 6] yielding 53 potential regional anesthesia techniques to be included.

### Delphi rounds

For this study, a three-round Delphi process was planned with two rounds of electronic voting through a web-based tool instrument (Google Forms, Google, Mountain View, California, USA) and a final round of mixed electronic voting and round table discussion consisting of teleconference using the Zoom platform (Zoom Video Communications). Selected experts were invited to participate in each stage of this consensus via mail, with a reminder sent after 1 week from the initial invitation. Members were not included in the panel if they did not participate in the first Delphi round after the reminder invitation. The steering committee set a 2-week duration for each round.

### Consensus achievement

As recommended by previous methodological papers [9], the consensus in our study was defined as ≥ 75% agreement between collaborators for inclusion and lower than ≤ 25% agreement for exclusion from the core curriculum list. Importantly, at each round, experts were invited to express their agreement, disagreement, or uncertainty regarding the inclusion of each technique in the core curriculum.

Regional anesthesia techniques with an agreement above 25% but below 75% at the first round were carried forward into a subsequent round. In this follow-up round, alongside presenting the outcomes and response frequencies from the first round, these techniques were readdressed for further consideration.

In the third round, the panel discussed techniques that failed to achieve consensus in earlier rounds. It was then decided to categorize techniques with agreement rates exceeding 50% as constituting a weak consensus for inclusion, while those with agreement rates ≤ 50% were deemed to represent a weak consensus for exclusion from the core curriculum.

## Results

The Delphi consensus took place from March to May 2024. Thirty experts were initially invited to participate in the consensus; however, four did not respond to the invitation leaving a total of 26 experts included in the Delphi consensus process.

**Fig. 1:**
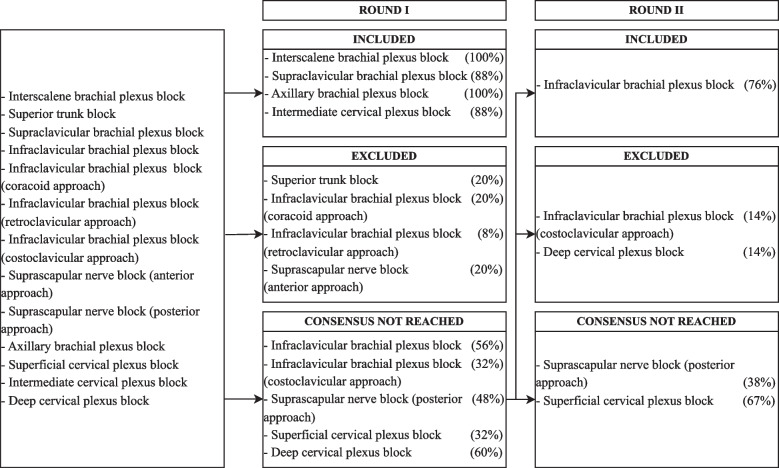
Process for the first two rounds of the Delphi method for upper limb blocks: Techniques that did not achieve a > 75% consensus in the second round were included with a weak consensus after further discussion in the third round of the Delphi process

**Fig. 2:**
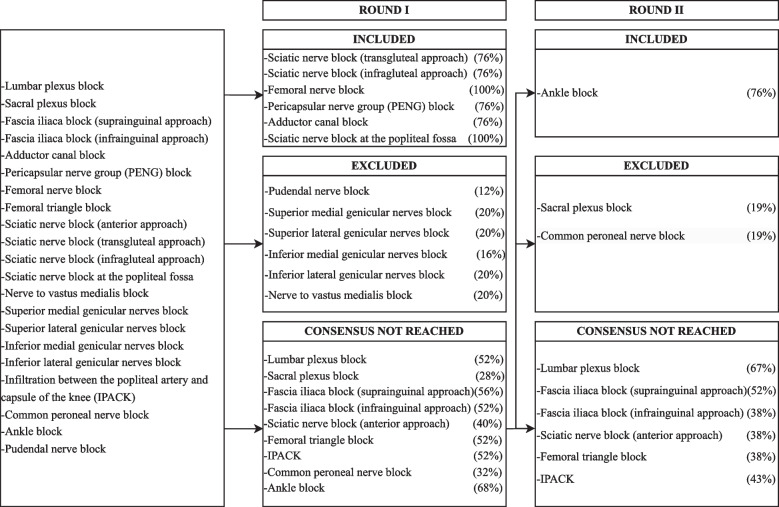
Process for the first two rounds of the Delphi method for lower limb blocks: Techniques that did not achieve a > 75% consensus in the second round were included with a weak consensus after further discussion in the third round of the Delphi process

**Fig. 3:**
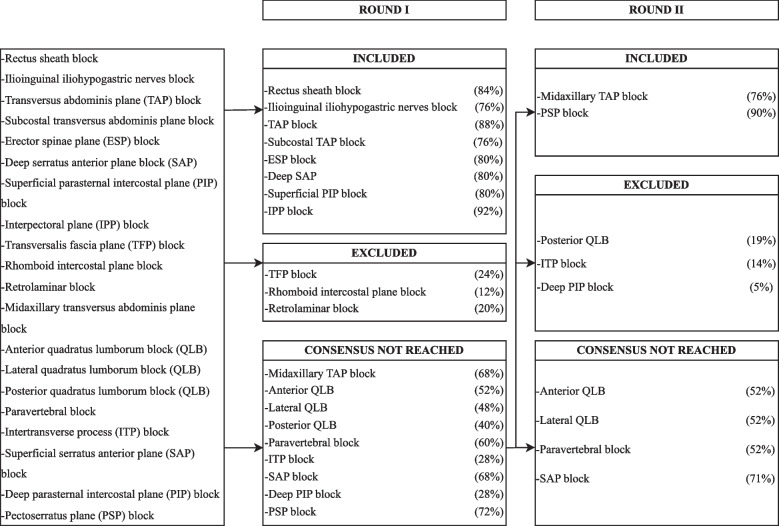
Process for the first two rounds of the Delphi method for paraspinal and fascial plane blocks: Techniques that did not achieve a > 75% consensus in the second round were included with a weak consensus after further discussion in the third round of the Delphi process

The flowchart of consensus achievement of the first and second round is depicted in Fig. 1 for the upper limb, in Fig. 2 for the lower limb, and in Fig. 3 for the paraspinal and fascial plane blocks.

At the end of the process, experts identified a total of 22 regional anesthesia techniques to be considered as core curriculum (five for the upper limb, seven for the lower limb, and ten for the paraspinal/fascial plane blocks) (Table 1). These techniques were approved in the third round of the Delphi process.

**Table 1:** Identified regional anesthesia techniques to be included in the anesthesiologists’ core curriculum

Included	
**Strong consensus**	**Weak consensus**
**Upper limb**	
1) Interscalene brachial plexus block 2) Supraclavicular brachial plexus block 3) Infraclavicular brachial plexus block 4) Axillary brachial plexus block 5) Intermediate cervical plexus block	1) Superficial cervical plexus block
**Lower limb**	
6) Femoral nerve block 7) Pericapsular nerve group block 8) Adductor canal block 9) Sciatic nerve block (transgluteal approach) 10) Sciatic nerve block (infragluteal approach) 11) Sciatic nerve block at the popliteal fossa 12) Ankle block	2) Lumbar plexus block 3) Fascia iliaca block (suprainguinal approach)
**Paraspinal and fascial plane blocks**	
13) ESP block 14) Deep SAP block 15) Superficial pectointercostal plane block 16) Interpectoral plane block 17) Pectoserratus plane block 18) Rectus sheath block 19) Ilioinguinal iliohypogastric nerves block 20) TAP block 21) Subcostal TAP block 22) Midaxillary TAP block	4) Anterior QL 5) Lateral QLB 6) Paravertebral block 7) SAP block

The regional anesthesia techniques have been divided in upper limb, lower limb, and fascial plane block techniques. *ESP* erector spinae plane, *SAP* serratus anterior plane, *TAP* transversus abdominis plane, *QLB* quadratus lumborum block

While a robust consensus was not initially reached for seven techniques (one for the upper limb, two for the lower limb, and four for the paraspinal/fascial plane blocks), it is noteworthy that a consensus exceeding 50% was achieved. Consequently, as decided in the third round of Delphi, it was deemed appropriate to include these techniques with a low consensus level, as delineated in Table 1.

## Discussion

Our research article delineates the outlines of an academic path for residents undergoing regional anesthesia training. We believe that his work is particularly significant given the absence of a national examination in Italy to assess residents’ proficiency upon completing their training. Establishing a national core curriculum for regional anesthesia techniques to be taught and performed during residency programs could help standardize the training and reduce variability among Italian anesthesia residents.

Proficiency in ultrasound-guided regional anesthesia requires the practitioners to acquire cognitive and technical skills; however, such skills are not easy to learn, and each different regional technique requires specific training with its proper learning curve [10–12].

Learning during residency could be facilitated by various educational instruments such as simulation, gamification, and through the use of constructive feedback and experts’ mentoring [13].

However, educational resources are finite, and various factors may restrict a practitioner’s ability to learn and master every technique delineated in the literature. Examples of such limitations include constraints on time, space, and even opportunities to apply acquired knowledge in real-life situations. For these reasons, this core curriculum could be of paramount importance in order to focus learning objectives and educational tools through the identified techniques.

In previous years, other researchers have dedicated their efforts to constructing a core curriculum tailored for regional anesthesiologists, particularly targeting the majority who may not pursue specialized fellowships in this field. For instance, in 2021, an international Delphi consensus was established [14]. However, despite similarities between our research articles, significant differences exist.

Firstly, the study by Chuan et al. [14] not only concentrated on defining the core curriculum but also explored training characteristics, competency assessments, and learning outcomes. Additionally, while the panel was international, Italian experts were minimally represented, comprising only 0.9% of the expert panel. It is essential to recognize that each country possesses distinct educational programs and healthcare resources. Therefore, we argue that it may not be entirely appropriate to transplant a core curriculum from one country to another without considering the unique attributes of each system.

Our study has some limitations that need to be addressed.

First, in our study, we did not involve the Italian scientific societies (i.e., the Italian Society of Anesthesia, Analgesia and Critical Care – SIAARTI and the European Society of Regional Anesthesia Italian Chapter – ESRA, Italian Chapter), and we recognized that engaging such societies in the core curriculum development could have both provided more strength to our recommendation and promoted a wider distribution; however, the panel of experts included most of the recognized experts in the field of regional anesthesia in Italy reducing such a bias. However, recognizing the importance of engaging these scientific societies, we hope that our work could be the first step in promoting a joint consensus for the development and/or the update of future national curricula.

Second, our study is based on a Delphi consensus based on electronic voting partially reducing the possibility of face-to-face or group interaction among participants, limiting the exchange of information.

## Conclusion

Experts recommend with strong consensus that 22 regional anesthesia techniques have to be considered as core curriculum in ultrasound-guided regional anesthesia (five for the upper limb, seven for the lower limb, and ten for the paraspinal/fascial plane blocks), while seven other techniques were included in the core curriculum with a low consensus.

This comprehensive curriculum aims to standardize training and ensure that residents acquire the essential skills required for effective and safe practice regardless of the residents’ subsequent specialization. By incorporating these techniques, educational programs can provide a structured and consistent approach to regional anesthesia, enhancing the quality of patient care and improving outcomes.

### Supplementary Information

Additional file 1: Supplementary Digital Content 1

## Data Availability

No datasets were generated or analysed during the current study.

## Abbreviations

ASRA: American Society of Regional Anesthesia
ESP: Erector spinae plane
ESRA: European Society of Regional Anesthesia
SAP: Serratus anterior plane
TAP: Transversus abdominis plane
QLB: Quadratus lumborum block
