# Supplementary material for: Anesthesiologists ultrasound-guided regional anesthesia core curriculum: a Delphi consensus from Italian regional anesthesia experts
Source: J Anesth Analg Crit Care. 2024 Aug 10;4:54. doi: 10.1186/s44158-024-00190-2 (PMC11316303; doi:10.1186/s44158-024-00190-2)
Supplement: Supplementary file 1 — Additional file 1: Supplementary Digital Content 1 [file 44158_2024_190_MOESM1_ESM.pdf]

Anesthesiologists Ultrasound-Guided Regional Anesthesia Core Curriculum: A Delphi consensus from Italian Regional Anesthesia Experts

Supplementary Digital Content 1

Search Strategy

**Pubmed**
**#1** ((("Anesthesia, Conduction"[MeSH]) OR ("Nerve Block"[MeSH]) OR "regional anesthesia"[Title/Abstract] OR "nerve block"[Title/Abstract]) AND ("nomenclature"[Title/Abstract] OR "terminology"[Title/Abstract] OR "classification"[Title/Abstract])) AND ("2019/01/01"[Date - Publication] : "2024/12/31"[Date - Publication])<br><br>**#2** (("Consensus"[MeSH] OR "Consensus Development Conference"[Publication Type] OR "Guideline"[Publication Type] OR "Position Statement"[Title/Abstract]) AND ("American Society of Regional Anesthesia and Pain Medicine"[Title/Abstract] OR ASRA[Title/Abstract] OR "European Society of Regional Anesthesia and Pain Therapy"[Title/Abstract] OR ESRA[Title/Abstract])) AND ("regional anesthesia"[Title/Abstract] OR "nerve block"[Title/Abstract] OR "upper limb block"[Title/Abstract] OR "lower limb block"[Title/Abstract] OR "fascial plane block"[Title/Abstract])) AND ("2019/01/01"[Date - Publication] : "2024/12/31"[Date - Publication])<br><br>**#3**: **#1** and **#2**
